# Which roads lead to access? A global landscape of six COVID-19 vaccine innovation models

**DOI:** 10.1186/s12992-024-01017-z

**Published:** 2024-03-26

**Authors:** Adrián Alonso Ruiz, Anna Bezruki, Erika Shinabargar, Kaitlin Large, Marcela Vieira, Iulia Slovenski, Yiqi Liu, Surabhi Agarwal, Anna Becker, Suerie Moon

**Affiliations:** 1 Global Health Centre, Graduate Institute of International and Development Studies, Chem. Eugène-Rigot 2, Genève, 1202 Switzerland; 2 Georgetown University, 3700 O St NW, Washington, DC, 20057 USA

**Keywords:** COVID-19, Vaccines, Global access, Equity, Global health, Pandemics, Innovation, Pharmaceuticals, Innovation models, Research and development, Public R&D funding

## Abstract

**Background:**

Unequal and inequitable access to Covid-19 vaccines in low- and middle-income countries (L&MICs) was a major political, ethical and public health failure in the pandemic. However, vaccine developers’ practices were not monolithic, but rather, took diverse approaches to supplying different countries, with important implications for global access.

**Results:**

Using data on R&D investments, regulatory approvals, manufacturing and purchase agreements, and vaccine deliveries, we identified six distinct innovation models that apply across the 14 COVID-19 vaccines with more international presence from 2020–2022. “Western Early Arrivers” Pfizer/BioNTech and Moderna supplied the largest volumes quickly and prioritized high-income countries (HICs) from registration to vaccine delivery. “Western Latecomers” Janssen and Novavax supplied intermediate volumes later, also prioritizing HICs but with a greater proportion to L&MICs. “Major Chinese Developers” Sinopharm and Sinovac supplied intermediate volumes early, primarily to middle-income countries (MICs). “Russian Developer” Gamaleya completed development early but ultimately supplied small volumes, primarily to middle-income countries (MICs). “Cosmopolitan Developer” Oxford/AstraZeneca supplied large volumes early to HICs and MICs at the lowest prices. Finally, “Small MIC Developers” CanSino, Bharat Biotech, Medigen, Finlay Institute and the Center for Genetic Engineering and Biotechnology (CGEB), exported relatively small volumes to a few MICs. Low-income countries (LICs) were not targeted by any developer, and received far fewer doses, later, than any other income group. Almost all developers received public funding and other forms of support, but we found little evidence that such support was leveraged to expand global access.

**Conclusions:**

Each of the six innovation models has different implications for which countries get access to which vaccines, how quickly, and at which prices. Each offers different strengths and weaknesses for achieving equitable access. Our findings also suggest that Western firms had the greatest capacity to develop and deliver vaccines quickly during the pandemic, but such capacity is rapidly becoming more globally distributed with MICs playing a significant role, especially in supplying other MICs. Given the critical role of public support in enabling pandemic vaccine development and supply, governments have both the capacity and responsibility to craft international rules that will make responses to future pandemics more equitable and effective.

**Supplementary Information:**

The online version contains supplementary material available at 10.1186/s12992-024-01017-z.

## Background

### Literature review and research gaps

Lack of access to COVID-19 vaccines during the height of the pandemic strained healthcare systems and economies, particularly in low- and middle-income countries (L&MICs), worsening existing health, social, and economic inequalities [1–6]. There is a substantial literature that seeks to explain why access was so globally unequal. For example, a significant body of scholarship has focused on advance purchase agreements (APAs) between vaccine developers and high-income countries (HICs) which allowed them to secure a large proportion of global supply early [7–29], or the lack of technology transfer to expand production and the contested role of intellectual property (IP) rights in impeding greater supply [7, 8, 12, 13, 15, 18, 22, 25, 26, 28–40]. Scholars have also highlighted regulatory and quality issues [8, 12, 17, 41, 42] and export restrictions [8, 17, 18, 20, 22, 25, 43] as important factors contributing to access inequities, although less frequently. There is also significant analysis of the limitations and challenges faced by COVAX, the vaccine pillar of the Access to COVID-19 Tools (ACT) Accelerator, pointing to competition with HICs for doses, lack of sufficient financing, and internal transparency and governance issues [8, 16, 17, 24, 26–33, 44–55].

The literature also discusses a range of policies that have been proposed or implemented to improve global access. Many scholars have argued in favor of waiving IP rights, together with the expansion of production capacities in L&MICs [7, 8, 10, 12–15, 18, 21, 22, 25, 26, 28, 30–33, 37, 39, 45, 46, 56]. Others argue for tying access conditions to public funding for pharmaceutical research and development (R&D), in light of the large amount of public investments in the development of many COVID-19 vaccines during the pandemic [18, 19, 21, 26, 36, 52, 57–61].

Scholars have also criticized certain measures, such as donations, for relying on a logic of charity that does not address structural causes of inequity, and a government’s use of vaccines to improve relations or increase political influence internationally, often referred to as “vaccine diplomacy” [13, 20, 22, 23, 27, 30, 31, 45, 46, 50, 53, 62].

The literature reviewed frequently presents vaccine developers as a more or less homogeneous group, with some important exceptions. For example, Wouters and collaborators [52] analyzed 26 vaccines in development or recently commercialized in early 2021, and the differential implications of each vaccine’s characteristics for global access; however, most of the vaccines evaluated were still in clinical development, the publication does not provide a picture of how these 26 vaccines were used [52]. Some scholars have differentiated vaccines developed in China or Russia, usually in the context of vaccine diplomacy [45, 46, 53, 63]. Suzuki and Yang (2022) analyzed the strategies followed by India, China, and Russia to provide global access to vaccines, but merges individual developers’ practices under the political and economic characteristics of each country of origin [64].

Therefore, there is not (to our knowledge) a comprehensive analysis of the different practices followed by COVID-19 vaccine developers and the implications for global access, nor a thorough picture of which developers contributed what, when and how to meeting global demand for vaccines. This study addresses these research gaps. Using data on R&D investments, global regulatory approvals, manufacturing and purchase agreements, and vaccine deliveries, we identify six distinct innovation models that cover the 14 COVID-19 vaccines with more international presence from 2020–2022. The next section provides background context on the R&D ecosystem for pandemic vaccines within which these innovation models are embedded.

### Pre-COVID-19 biosecurity R&D system

There is a distinct niche in the broader pharmaceutical R&D ecosystem that focuses on developing medical products to address biological threats to security (including Emerging Infectious Diseases, EIDs), characterized by three key features. First, the unpredictability of emerging infectious disease outbreaks discourages companies and investors from investing in R&D for these diseases [65–67]. This has led to a greater role of the public sector in different countries, with a range of national institutions and funding mechanisms involved across the entire development chain [68, 69]. The public sector accounted for 77% of the global R&D investments for EIDs from 2014 to 2019 [70]. This feature has contributed to an unstable funding landscape that follows “cycles of panic and neglect”, where funding increases during outbreaks, and then rapidly declines when the immediate threats subside [65–67].

Second, R&D investors and recipients are heavily concentrated in the United States, which disbursed 63.28% (USD 3.4 billion) and received 54.08% (USD 2.9 billion) of all investments during this time period [69]. Many countries do not have dedicated institutions or policies to develop health products for potential pandemics [70].

Third, the biosecurity R&D system is oriented towards national security, historically linked to the protection of military and civilian populations from biological threats. Such systems are not limited to Western countries, as India, China, Russia, or Cuba have all established their own R&D ecosystems to address disease outbreaks with different degrees of connection to the military [63, 68, 71–74]. This national security orientation of R&D means that global access to resulting technologies has not been a priority.

In 2017, the creation of the Coalition for Epidemic Preparedness Innovations (CEPI) aimed to address the chronic lack of funding for vaccine R&D for EID and to improve equitable access to such vaccines globally. CEPI operates by channeling funding from donor countries and philanthropies to vaccine developers, while linking the investment to a set of access principles and conditions [75]. Between 2020 and 2021, CEPI received funding from 24 actors, mostly governments (the top 5 investors were the UK, Germany, Norway, Saudi Arabia and the European Commission).

The three characteristics mentioned above shaped the R&D response to the COVID-19 crisis. The unprecedented surge in investments to de-risk and expedite the development of new vaccines from preclinical to clinical stages mostly came from public sources. The US government was the largest investor in vaccine R&D between 2020 and 2021 (over USD 2.2 billion), followed by the German government (USD 1.5 billion), CEPI (USD 1.4 billion and mostly funded by public sources), the UK government (USD 500 million), and the European Union (USD 331 million) [76]. As a comparison, between 2007 and 2022, average annual investments on vaccine R&D for malaria, tuberculosis and HIV combined are USD 1.1 billion [70]. Governments prioritized investments in national developers, whereas CEPI was the funder with the most geographically diverse investment portfolio. If we include APAs concluded before a vaccine received regulatory approval (mostly made by the US and EU) as pull incentives for R&D, as they de-risked investments in clinical trials and expanding manufacturing capacity, the total public sector contributions to R&D increase to USD 51.1billion [76].

Thus, during the Covid-19 crisis, large amounts of public funding paid for the development and production of vaccines, first to address national needs and only subsequently to address global demand.

## Methodology

### Sources of data

We used two data sources to generate our research findings. First, we used UNICEF’s COVID Market Dashboard [77], an open platform with data on the COVID-19 vaccine market. We accessed the data related to COVID-19 vaccines’ registration status (Additional file 1), prices (Additional file 2), production (Additional file 3), purchases (Additional file 4), and deliveries (Additional file 5) for all vaccines tracked between January 2020 and December 2022. Second, we used the COVID-19 vaccine R&D investments tracker from the Geneva Graduate Institute’s Global Health Centre [76], which tracked announcements of investments between early 2020 and July 2021 (Additional file 6).

### Selection of vaccines

**Fig. 1:**
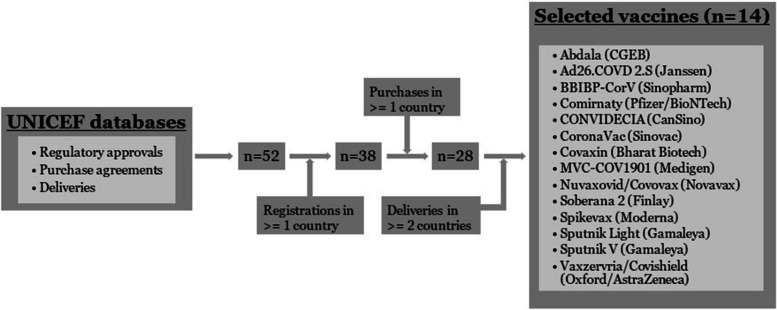
Selection process

We selected the vaccines that contributed to international supply (i.e. were exported). We began with all 52 COVID-19 vaccines listed in UNICEF’s databases. We then excluded 28 vaccines based on the lack of data on regulatory approvals (14 exclusions) or purchase agreements (14 exclusions). Finally, we excluded an additional 10 vaccines that did not have evidence of being delivered in two or more countries. As a result, we selected 14 vaccines for further analysis (Fig. 1).

### Development and analysis of the six innovation models

We performed a descriptive analysis of R&D investments received, vaccine approvals, production and purchase agreements, vaccine prices, and deliveries. We used median time as a measure of centrality to analyze the timelines of vaccine registrations and purchases, as both presented a right-skewed distribution.

The development of the six models followed an inductive approach based on the analysis of the variables shown in Table 1 for each vaccine assessed. Data on vaccine prices was used to provide a broader context and deeper understanding of companies’ practices, but were not used to develop the models, given the limited data availability.

**Table 1:** Variables analyzed to create the typology

Feature studied	Variable studied per vaccine
R&D investments	R&D funder’s country of origin
	Investment size
Regulatory approvals	Number of regulatory approvals
	Percentage of regulatory approvals by country’s income level
	Time to regulatory approval since the first approval globally
Manufacturing agreements	Number of manufacturing agreements
	Percentage of manufacturing agreements by country’s income level
Purchase agreements	Number of doses committed
	Percentage of doses sold by purchaser’s income level
	Timeline of agreements since each vaccine’s first purchase
Vaccine deliveries	Number of doses delivered
	Percentage of deliveries by recipient’s income level
	Timeline of deliveries by recipient’s income level

In the analysis of registrations, we inductively distinguished between “early arrivers” and “latecomers” after observing a natural separation between developers that managed to obtain regulatory approvals in 25% of all the countries where they are registered before the median time for all registrations (228 days).

Based on these variables, we devised the following typology of vaccine developers’ innovation models:

1. **Western Early Arrivers:** Pfizer/BioNTech and Moderna. Characterized by the substantial public investments received (through push and/or pull mechanisms), their ability to reach global markets quickly and in large volumes, and their prioritization of HIC markets.
2. **Western Latecomers:** Janssen and Novavax. Characterized by the relatively smaller amounts of public funding received, slower entry to global markets, intermediate volumes supplied, and larger focus on L&MICs compared to Western Early Arrivers.
3. **Major Chinese Developers:** Sinopharm and Sinovac. Characterized by their early arrival to the global market, prioritization of middle-income countries (MICs), and the intermediate volumes supplied.
4. **Russian Developer:** Gamaleya’s two vaccines Sputnik V and Sputnik Light. Characterized by their focus on middle-income countries (MICs), their creation of a wide network of manufacturing partners, and low volumes supplied.
5. **Cosmopolitan Developer:** Oxford/AstraZeneca. Characterized by substantial funding from public sources and CEPI, and its capacity to supply and reach global markets quickly and in large volumes, with no apparent prioritization between HICs, UMICs, and LoMICs.
6. **Small Middle-Income Country (MIC) Developers:** CanSino, Bharat Biotech, Medigen, Finlay Institute and the Center for Genetic Engineering and Biotechnology (CGEB). Characterized by low volumes supplied internationally to a relatively small number of L&MICs and with the exception of CanSino and Bharat Biotech, their late arrival to the market.

### Limitations and areas for further research

Our analysis is limited by its reliance on publicly available datasets, since a substantial portion of the relevant data on this topic have not been made publicly available. Nearly 41% (40.94%) of the almost 16 billion doses delivered tracked in UNICEF’s database did not include information about which vaccine was delivered. The dataset does not contain information on the vaccines rolled out in China, Germany, Russia, or Cuba, which likely skewed the results relevant to these countries. In particular, given the large populations of China and Russia, and with several of the vaccines analyzed developed in both countries, our findings most likely under-represent the total global volume of vaccines supplied by Chinese and Russian developers. Further research and transparency are needed to understand vaccine deliveries in these countries.

The data on vaccine R&D investments only captures public announcements, limiting the capacity to compile information on actual disbursements or private investments. In addition, the content of the agreements, including purpose (R&D activities covered), access conditions, and type of investment (e.g., loan, grant, equity) are often unavailable [76].

Similarly, the reliance on publicly available data for the manufacturing and purchase agreements pose another limitation, as these agreements are often confidential. Data on vaccine prices in particular is very limited, as most countries and developers have not disclosed prices. These limitations reduce the granularity of the analysis and potentially skew the results, if the available data analyzed is not adequately representative. Increased transparency and further research would be needed to understand the implications of these contracts and agreements for vaccine innovation and access.

Using the World Bank’s income level classification presents some technical limitations such as the lack of updated data for some countries (e.g., Venezuela). In addition, by merging heterogeneous countries into single categories, a level of detail is lost regarding the regional patterns across income groups [78]. Additionally, further relevant research could be done analyzing the distribution of these 14 vaccines by geographical classification.

Finally, this publication does not include the perspective of vaccine developers regarding their own innovation models and strategies, which limits understanding of their decisions. Further research on the factors that drove each developer’s internal decision-making during the crisis would be useful.

## Results

### Description of vaccine developers and their vaccines

The group of selected developers includes different types of organizations: nine publicly listed companies, two non-listed private companies, six research or academic centers, and one state-owned company. Partnerships are diverse as well, occurring between publicly listed companies (Pfizer and BioNTech), academic institutions and publicly listed companies (Oxford University and AstraZeneca) or non-listed companies (Bharat Biotech and the Indian Council of Medical Research). Vaccine developers are based in HICs such as the US or Europe as well as in MICs such as China, Cuba, Russia, or India.

The vaccines have a diverse range of platforms. Innovative technologies such as adenoviral vectors (Oxford/AstraZeneca, Janssen, Gamaleya, CanSino) and mRNA platforms (Pfizer/BioNTech, Moderna) coexisted with traditional platforms, such as protein subunit vaccines (Medigen, Finlay Institute, CGEB, Novavax), or whole inactivated viruses (Bharat Biotech, Sinovac, Sinopharm). This has implications for access because the logistic requirements for some vaccines are heavy, for example, mRNA vaccines usually require cold chain and ultra-cold chain logistics, compared to other vaccine platforms [79] (Table 2).

**Table 2:** Characteristics of vaccine developers and vaccines analyzed

**Developer**	**Vaccine**	**Organizational form**	**WHO EUL**^**a**^ [80] **date and NRA**^**b**^** of record**	**Vaccine platform**	**Doses**^**c**^	**Vaccine performance**^**d**^	**Storage requirements**	**Shelf life (at 2–8ºC)**
Medigen Vaccine Biologics Corp	MVC-COV1901	Publicly listed company	EOI submitted	Recombinant spike protein vaccine [81]	2 [82]	NA^e^	2–8 °C [82]	12 months [82, 83]
Pfizer/BioNTech	Comirnaty	Publicly listed company	31 December 2020 (EMA)	Mrna - LNPs [84]	2 [84]	91% [84]	-25 - -15°[79]	1 month [84]
Finlay Institute	Soberana 2	Public research center	No	Recombinant RBD vaccine [85]	2 [85]	69.7% [85]	2–8 °C [85]	4 months [86]
Oxford/AstraZeneca	Vaxzervria/Covishield	Academic/Publicly listed company	Vaxzevria: 15/04/2021 (EMA) Covishield: 15/022021 (CDSCO)	Chimpanzee adenoviral vector (ChadOx1) [87]	2 [88]	74% [88]	2–8 °C [88]	6 months [79]
Center for Genetic Engineering and Biotechnology	Abdala	Public research center	Dossier accepted for review	Recombinant RBD vaccine [89, 90]	3 [89, 90]	92.3% [89, 90]	2–8 °C [89, 90]	6 months [89, 90]
CanSino Biologics	CONVIDECIA	Publicly listed company	19/05/2022 (NMPA)	Adenoviral vector-based vaccine (Ad5) [91]	1 [91]	58% [91]	2–8 °C [91]	12 months [92]
Bharat Biotech International Ltd./Indian Council of Medical Research	Covaxin	Nonlisted company/ Public research center	03/11/2021 (CDSCO) – Suspension of supply	Whole virion inactivated vaccine [93]	2 [94]	78%^f^ [94]	2–8 °C [94]	9 months [93]
Novavax, Inc./Serum Institute of India	Nuvaxovid and Covovax	Publicly listed company/Nonlisted company	Covovax: 17/12/2021 (CDSCO) Nuvaxovid: 20/12/2021 (EMA)	Protein subunit -- Nanoparticle vaccine [95]	2 [95]	90% [95]	2–8 °C [95]	6 months [95]
Gamaleya Research Institute of Epidemiology and Microbiology	Sputnik Light	Public research center	No	Human Adenoviral vector-based vaccine (Ad26) [92]	1 [92]	92% [92]	-18ºC [92]	3 months at -18ºC [92]
Gamaleya Research Institute of Epidemiology and Microbiology	Sputnik V	Public research center	Process restarted. Dossier accepted for review	Human Adenoviral vector-based vaccine (Ad26 and Ad5) [96]	2 [96]	92% [96]	-18ºC [96]	3 months at -18ºC [92]
Sinovac Biotech Ltd	CoronaVac	Publicly listed company	01/06/2021 (NMPA)	Whole virion inactivated vaccine [97]	2 [97]	51%, 65.3% and 83.5%^g^ [97]	2–8 °C [97]	24 months [92]
Moderna, Inc	Spikevax	Publicly listed company	30/04/2021 (EMA)	mRNA - LNPs [98]	2 [99]	94% [99]	-15 °C to -25ºC; [99]	30 days [99]
Janssen Pharmaceuticals	Janssen - Ad26.COV 2.S	Publicly listed company	12/03/2021 (EMA)	Human Adenoviral vector-based vaccine (Ad26) [100]	2	One dose^h^: 67% Two doses: 75% [100]	-20ºC [100]	11 months [100]
Sinopharm – Beijing Institute of Biological Products Co., Limited	BBIBP-CorV	State-owned company	7/05/2021 (NMPA)	Whole virion inactivated vaccine [101]	2 [101]	79% [101]	2–8 °C [101]	24 months [101]

^a^Emergency Use Listing

^b^National Regulatory Agency

^c^Excluding booster doses

^d^Data retrieved, vaccine efficacy against symptomatic (mild, moderate or severe) COVID-19 (Alpha variant), from Phase 3 clinical trials

^e^Data available relates to non-inferiority against Oxford/AstraZeneca. These were not included in the present table

^f^With Delta variant predominant

^g^From clinical trials in Brazil, Indonesia and Turkey

^h^In moderate to severe-critical COVID-19 for both one dose and two dose

### R&D investments

**Fig. 2:**
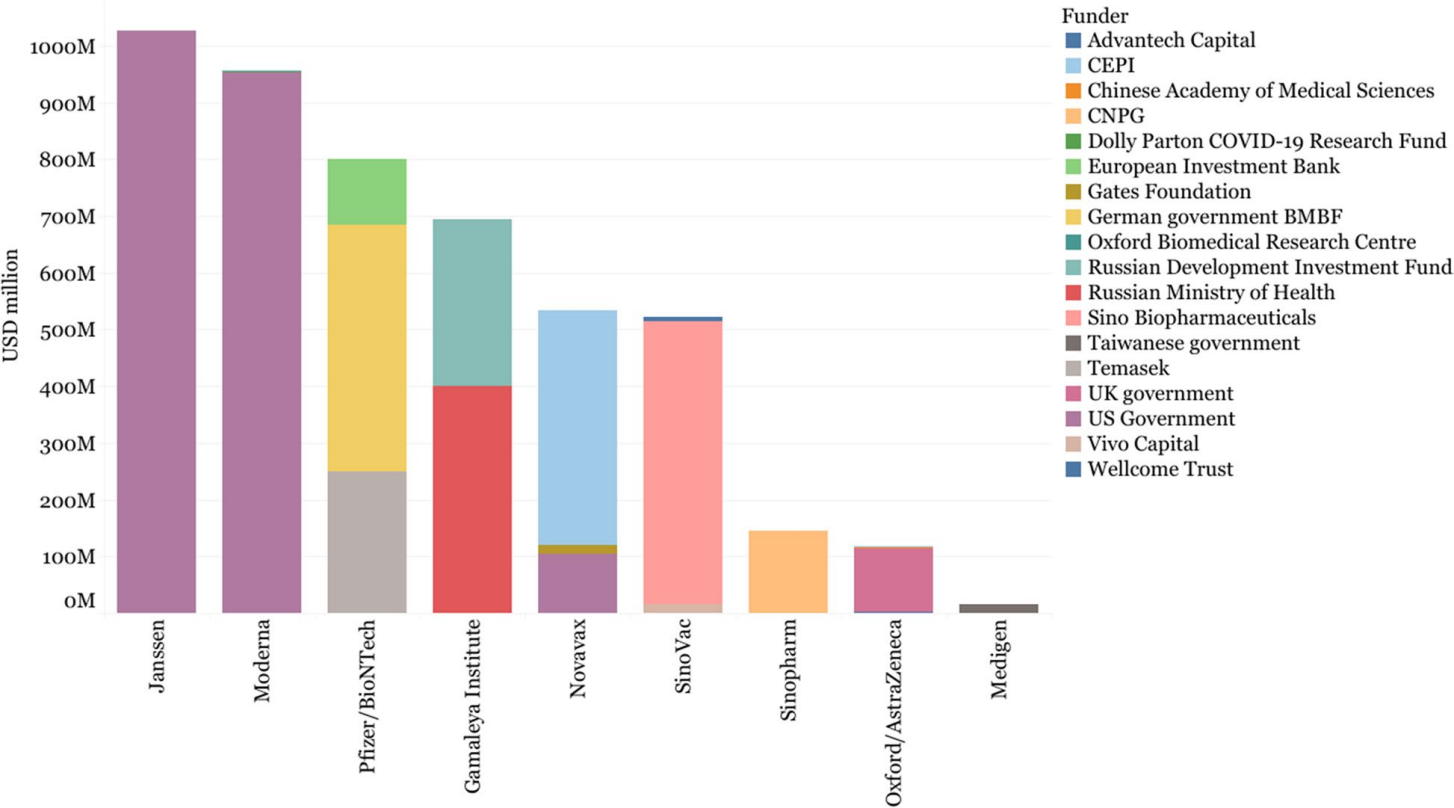
R&D investments for the selected COVID-19 vaccine developers

**Fig. 3:**
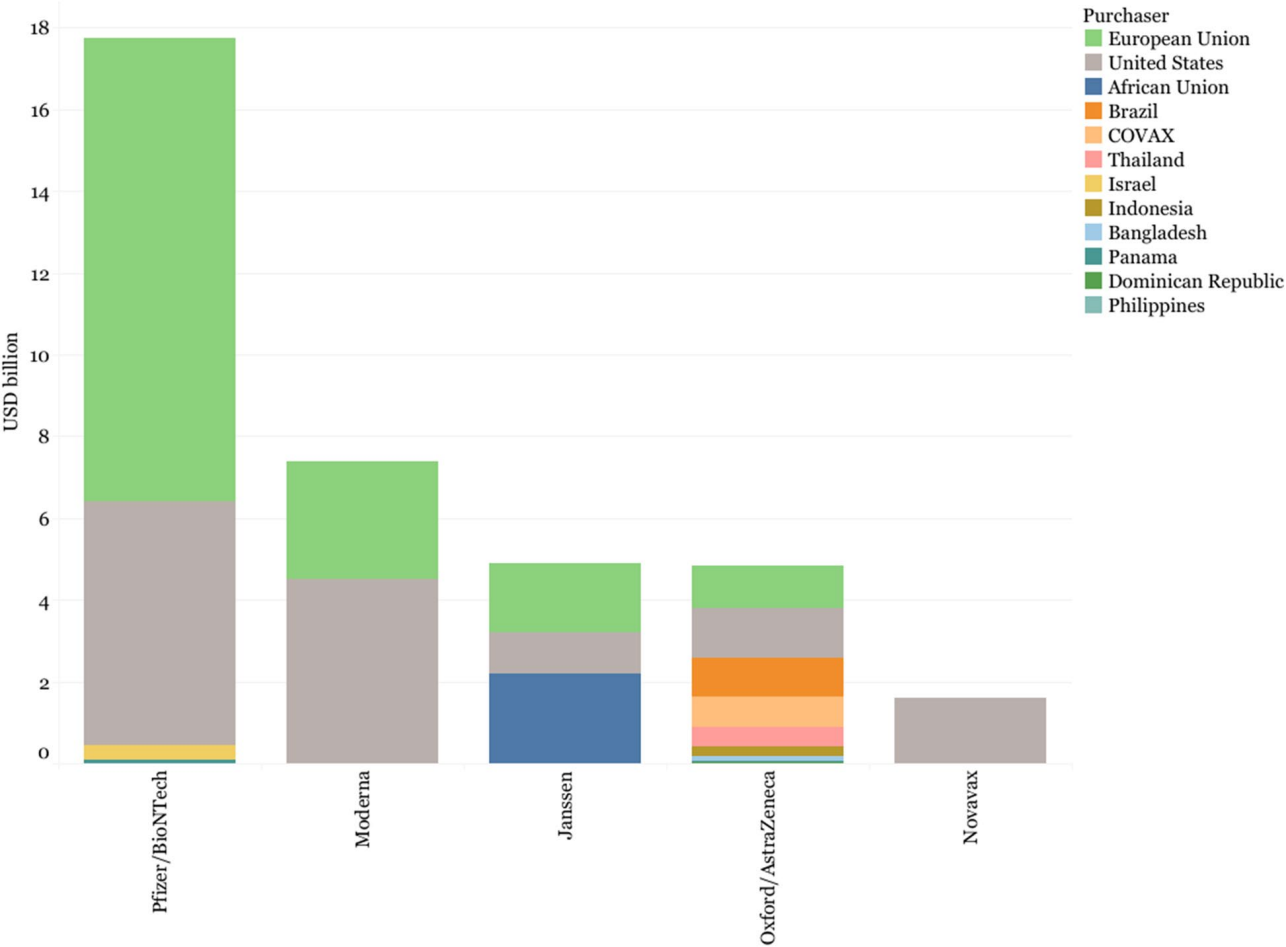
Value of declared advance purchase agreements of selected COVID-19 vaccines

Out of the 14 vaccines selected for analysis, there is evidence of public investments for 12 of them, with CanSino and Sinovac the exceptions. However, there are notable differences between Western and non-Western developers in the type and amount of support received to expedite preclinical and early clinical development (Fig. 2). These differences increase when considering APAs, especially with Western Early Arrivers signing APAs with the US and the European Union. Notably, Pfizer did not receive public grants but its partner BioNTech did receive public grants and loans. The Pfizer/BioNTech vaccine also benefited from publicly-financed APAs, see Figs. 2 and 3 below. In contrast, Janssen and Novavax received funding from other sources, such as the APA between Janssen and the African Union or the agreement between Novavax and COVAX, the global platform tasked with purchasing and delivering COVID-19 vaccines globally. Oxford/AstraZeneca’s APA with CEPI/COVAX represents a large portion of the public support received.

There is limited evidence about the role of the public sector in funding and facilitating the development of most non-Western COVID-19 vaccines. Both Russian vaccines, Sputnik V and Sputnik Light, were supported by the Russian Direct Investment Fund (RDIF) with at least USD 295 million, as well as by the Russian Ministry of Health (USD 399.5 million) [102, 103]. In addition, the Russian Ministry of Defense supported the development of preclinical and clinical trials [71]. There is little publicly-available information about the economic support provided by Chinese institutions, except for the approximately USD 142 million allocated by the state-owned company Sinopharm to the development of two inactivated COVID-19 vaccines [76]. Sinovac funded the development of its vaccine through an acquisition deal with Sino Biopharm, a Hong Kong based company that acquired 15.03% of Sinovac in December 2020; and through securing USD 15 million in investments from private investors.

Bharat Biotech’s Covaxin was developed in partnership with the Indian Council of Medical Research (ICMR, the main public agency in India for the coordination and promotion of biomedical research) [104] and received funding to expand manufacturing capacity from the Indian Government [105]. Medigen’s vaccine received in-kind support from the US NIH (biological samples to test its vaccine in animals), and received public subsidies from the Taiwanese government to run a Phase I clinical trial [106, 107]. The two Cuban vaccines, Abdala and Soberana 2, were developed in public research centers that are part of the state-owned conglomerate BioCubaFarma [108, 109], although there is no publicly available data on the costs of development and the resources invested by the Cuban government.

In summary, there is evidence of public funding for the development of all vaccines analyzed (including those developed within public institutions), except for Sinovac and CanSino.^1^ The evidence available points towards a higher monetary level of support for Western developers, especially when considering the use of APAs, which fostered the late-stage development of several vaccines. Nevertheless, the lack of publicly available data prevents us from painting a comprehensive picture of the role of public and private funding in the development of all vaccines.

### Vaccine regulatory approvals

Regulatory approvals can provide some insight into the priorities of vaccine developers. That is, we can look at the countries and regions where developers first achieved regulatory approval, as well as the overall number of places where they filed for registration, to get a sense of their priority markets and the extent of their capacity and/or interest in providing global access. Admittedly, this is an imperfect indicator of a developer’s strategy, particularly if used alone, given that the timeline between the submission of a regulatory dossier and approval depends largely on the regulatory agency. Nevertheless, it remains a useful indicator, particularly given the urgency of the pandemic which led to the use of emergency use authorizations and rolling review processes that fast tracked approvals, likely facilitating developers to file for registration for their products globally [110].

**Fig. 4:**
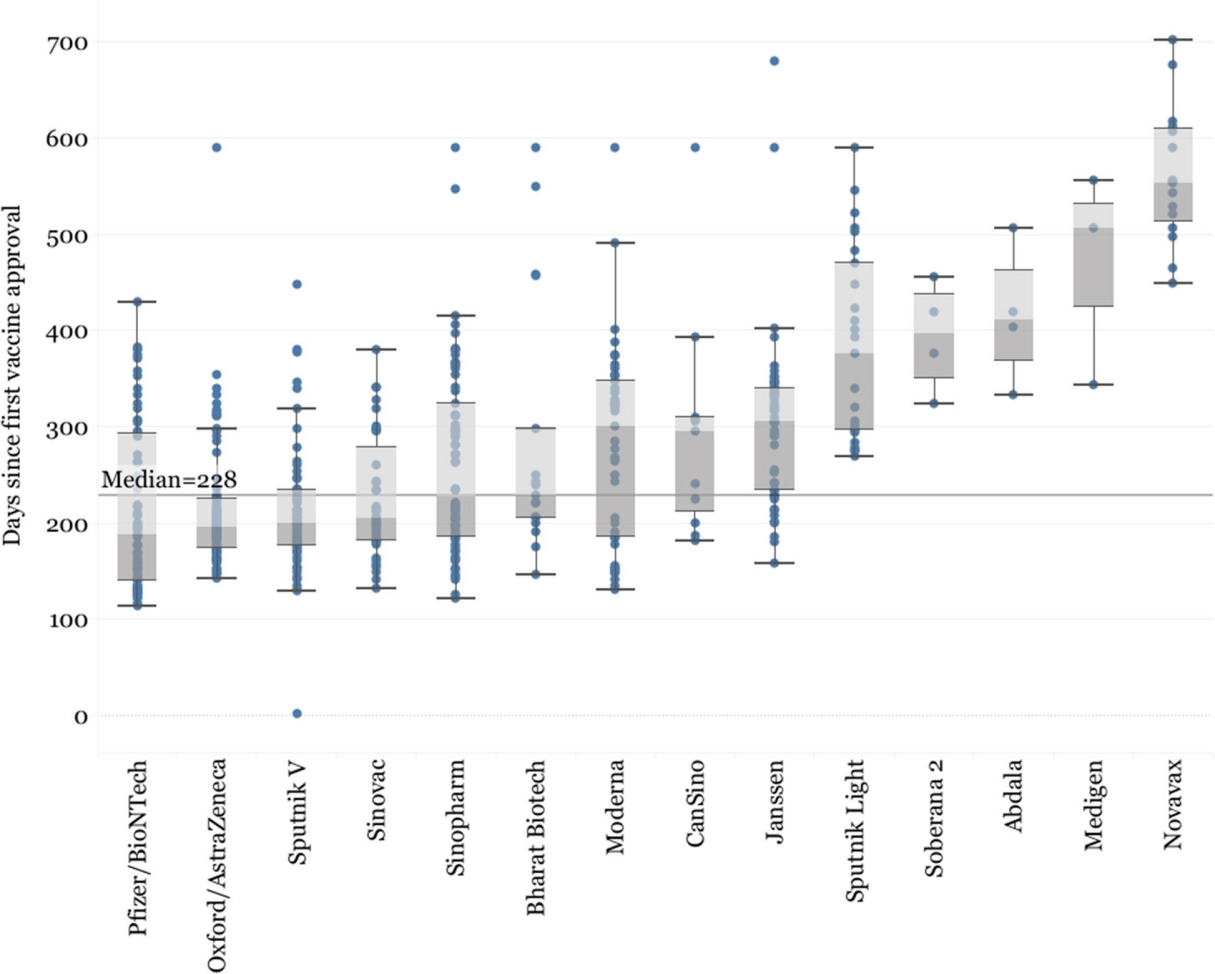
Distribution of regulatory approvals for each vaccine. The Y-axis represents days since the approval of the first vaccine globally (Sputnik V in Russia)

An analysis of the regulatory approvals timeline shows a separation in two groups of vaccine developers, based on the speed to obtain regulatory approval globally (Fig. 4): “early arrivers” (Pfizer/BioNTech, Moderna, Sinovac, Sinopharm, Oxford/AstraZeneca, Sputnik V, Bharat Biotech, and CanSino), which obtained at least 25% of all their regulatory approvals before 228 days (or roughly 7.5 months, the median time of all developers together) and “latecomers” (Novavax, Soberana, Abdala, Medigen, Sputnik Light and Janssen), which obtained all of their approvals after 228 days. The exception is Janssen, which is situated closer to the early arrivers, with 25% of its approvals obtained in 233.5 days, but still above the median threshold of 228 days.

**Fig. 5:**
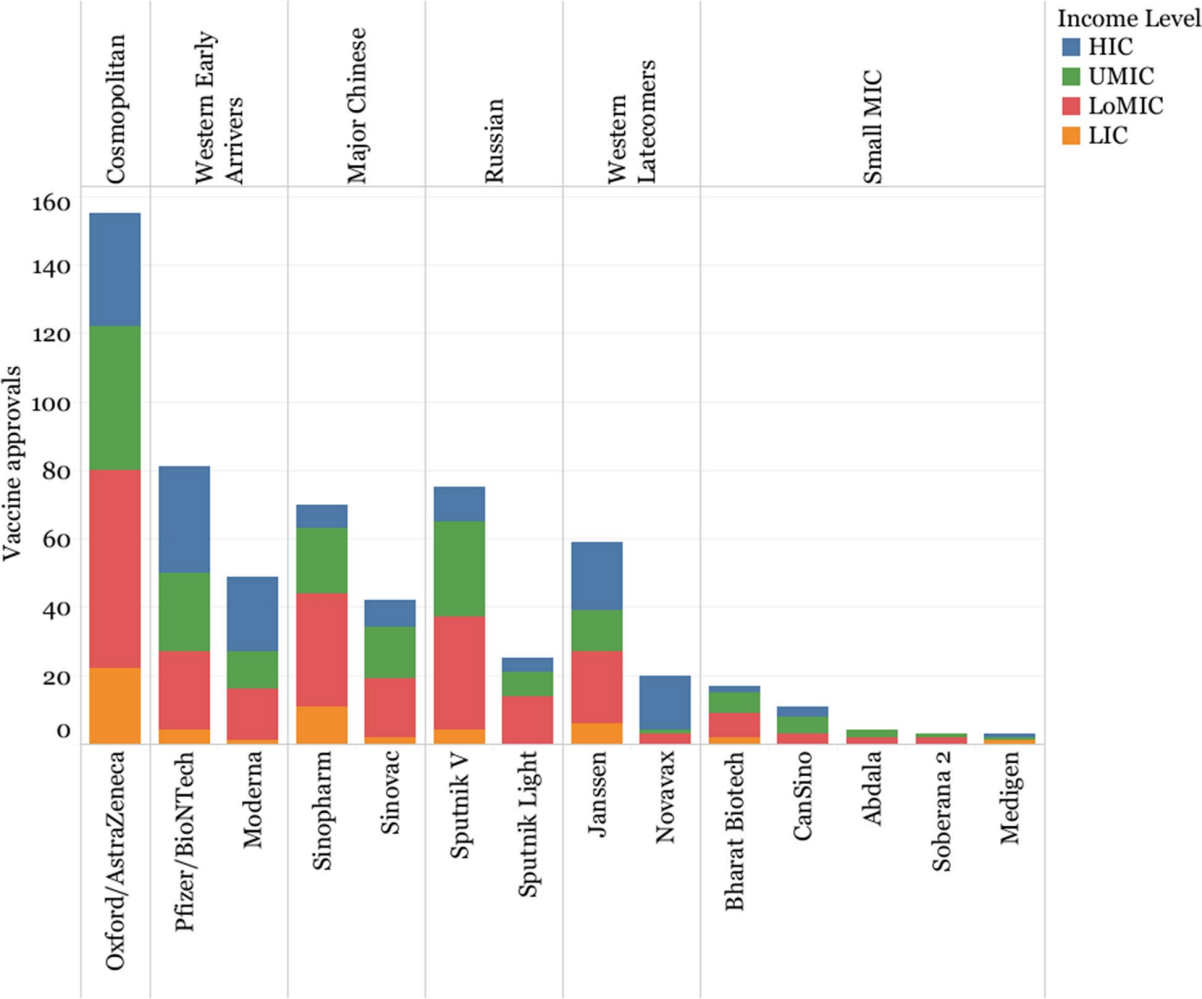
Total number of vaccine approvals by innovation model and income level

Oxford/AstraZeneca simultaneously had the largest number of global registrations and was the fastest to obtain all of its regulatory approvals, obtaining 75% of all its regulatory approvals in 224 days after the approval of the first vaccine (Fig. 5). Novavax, on the other hand, was the last in the group to achieve its first approval, which came 448 days after the approval of the first vaccine globally, Sputnik V in Russia.

Most vaccines we analyzed obtained WHO Emergency Use Listing (EUL), which was necessary to enter into purchase agreements with UNICEF or COVAX. Those that had not received EUL (to date) were Medigen, which submitted an expression of interest, Abdala and Sputnik V, which had been accepted for review, and Sputnik Light and Soberana 2, which had not submitted an expression of interest (Table 2). All Western developers (including Oxford/AstraZeneca) except Novavax obtained WHO EUL within the first four months of 2021. In contrast, Bharat Biotech, Sinopharm, Sinovac, and CanSino obtained EUL in the second half of 2021.

When regulatory approvals are sorted by country income levels, we observe a clear divide in market prioritization among the developers (Fig. 5). Whereas Western developers predominantly obtained approvals in HICs, Major Chinese and Russian Developers focused more on UMICs and LoMICs. However, this broad trend becomes a bit more nuanced when looking at individual companies. For example, out of the 20 countries that granted regulatory approval to Novavax, 80% were HICs, more than Janssen (33.90%, *n* = 59), Pfizer/BioNTech (38.27%, *n* = 81), and Moderna (44.90%, *n* = 49). Sinopharm (*n* = 70), Sputnik V (*n* = 75), and Sinovac (*n* = 42), obtained a higher proportion of approvals in LoMICs (47.14%, 44%, 40.48%) than UMICs (27.14%, 37.33%, 35.71%). Oxford/AstraZeneca (*n* = 155) had a more even distribution of approvals, with the highest number of approvals in LICs (*n* = 22, 14.19%), which were otherwise deprioritized by all other developers.

### Creating manufacturing networks

By examining data on the manufacturing networks formed by vaccine developers, we can explore the production needs and capacities of developers during the pandemic, and get a sense of longer-term strategies.

**Fig. 6:**
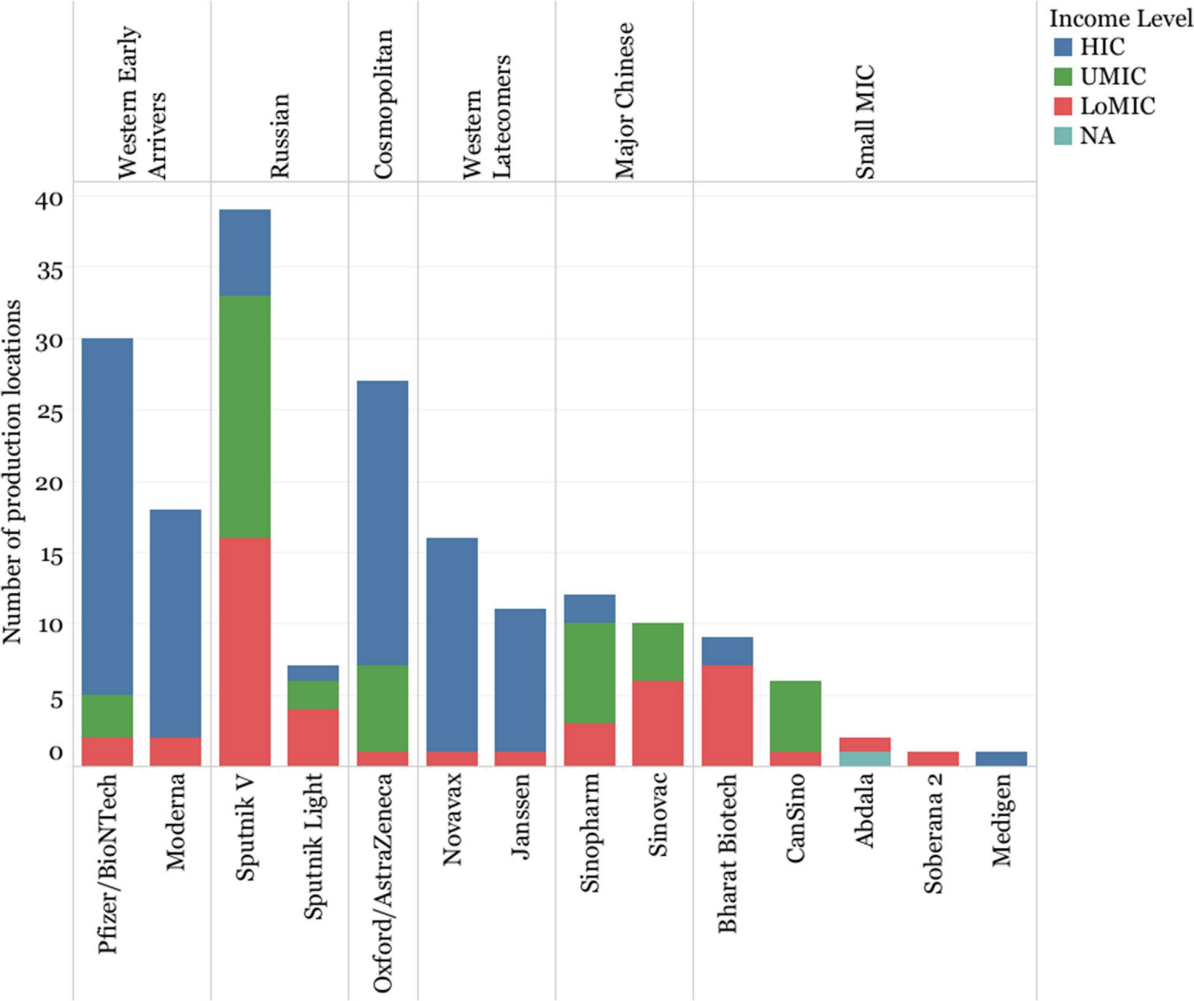
Number of manufacturing agreements by income level of the manufacturing partner’s country

For instance, Western developers and Oxford/AstraZeneca prioritized manufacturing agreements with companies based in HICs (86.19% of their agreements on average). On the other hand, the Russian and Major Chinese Developers frequently focused their activities in LoMICs and UMICs (on average, 45.79% with LoMICs and 42.62% with UMICs). The rest of the developers signed fewer agreements (Fig. 6).

Fill-and-finish agreements were the agreements most frequently announced (37.04% of all agreements). Western developers and Oxford/AstraZeneca also signed several agreements to produce drug substance (24.39% on average). Neither Sinopharm nor Sinovac reported agreements to produce drug substance or adjuvants, and instead rely on fill-and-finish agreements (68.18% of all their agreements).

**Fig. 7:**
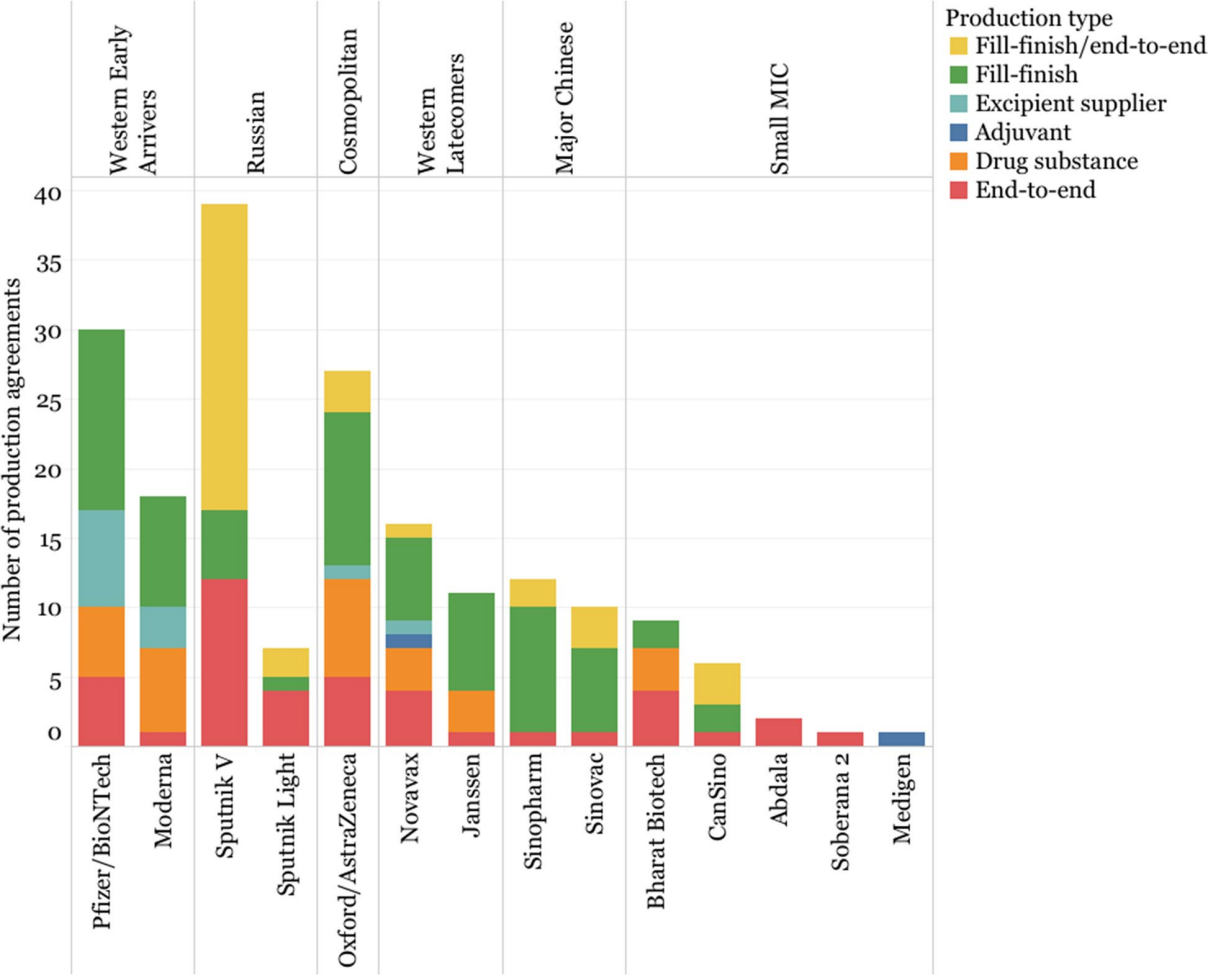
Type of manufacturing agreements

Gamaleya built the largest network of manufacturers in the group to produce Sputnik V, with 39 different manufacturers, 56.41% of which intended to begin as fill-and-finish, and then progress to end-to-end production agreements and 30.77% categorized as end-to-end. Nevertheless, it is unclear how many of these agreements led to actual production, given the low volume of deliveries and the production and quality issues surrounding the development of Sputnik V [102]. In addition to Gamaleya, Bharat Biotech, Novavax, and both Cuban developers also signed at least 25% of all their agreements as end-to-end agreements, which could potentially signal a lack of in-house capacity to produce large volumes of their vaccines and/or a greater willingness to engage in technology transfer [64] (Fig. 7).

### Signing purchase agreements

**Fig. 8:**
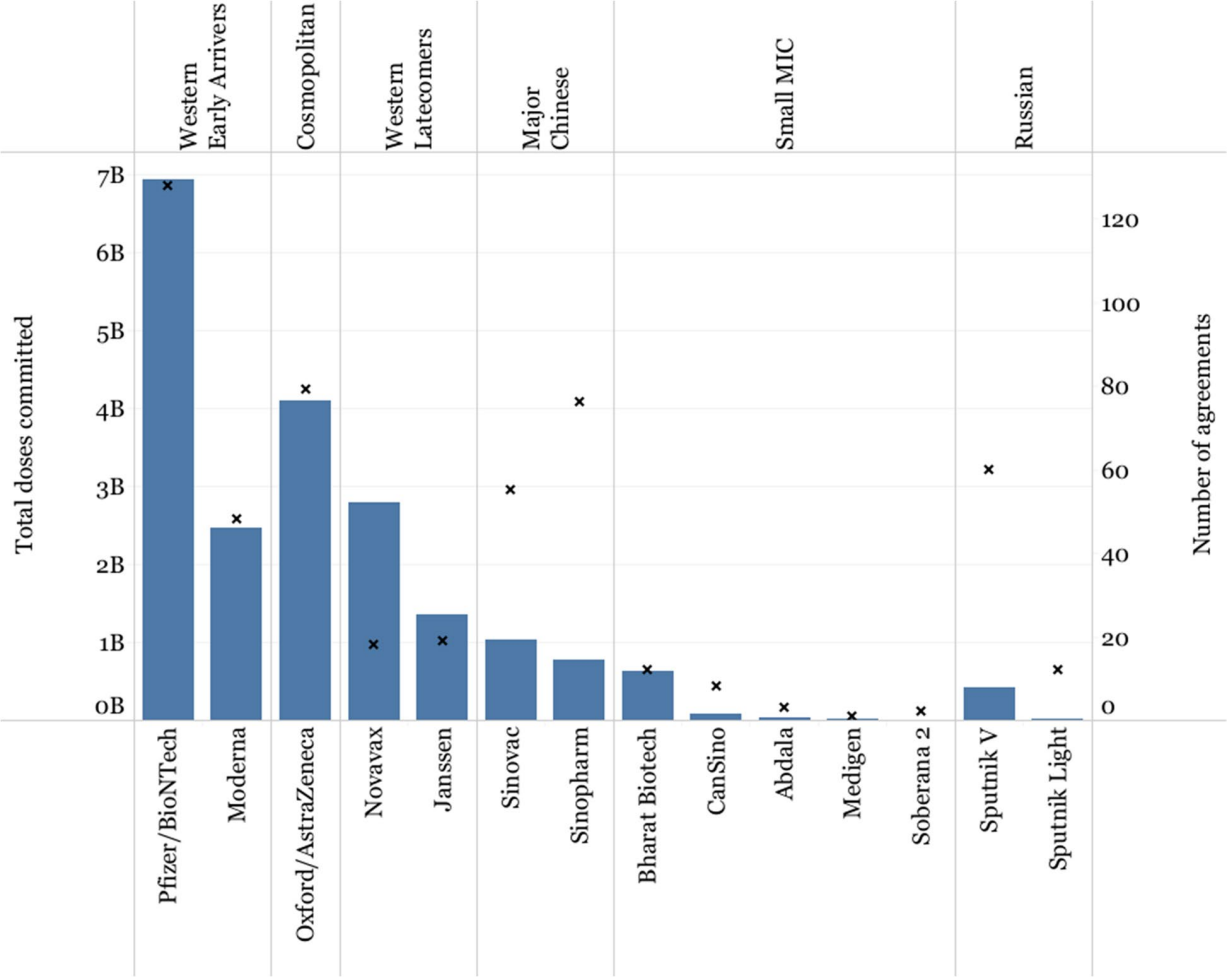
Doses committed and purchase agreements. Left axis (bar chart) shows the number of doses committed. Right axis (X’s) shows the number of purchase agreements

The number of purchased doses by countries can provide an idea of the supply capacity of the different developers and their priority markets. For example, although Sinopharm and Gamaleya were among the developers with the highest number of agreements, they committed fewer doses than Western developers and Oxford/AstraZeneca, potentially illustrating the differences in supply and production capacity of these two groups (Fig. 8).

Whereas Western Early Arrivers committed the largest share of their doses to HICs (66.84% Pfizer/BioNTech and 79.59% Moderna), Western Latecomers showed a different pattern, with Novavax committing 48.33% of its doses to COVAX (and 45.35% to HICs) and Janssen committing 29.95% of its doses to LoMICs through its APA with the African Union (and 47.46% to HICs).

**Fig. 9:**
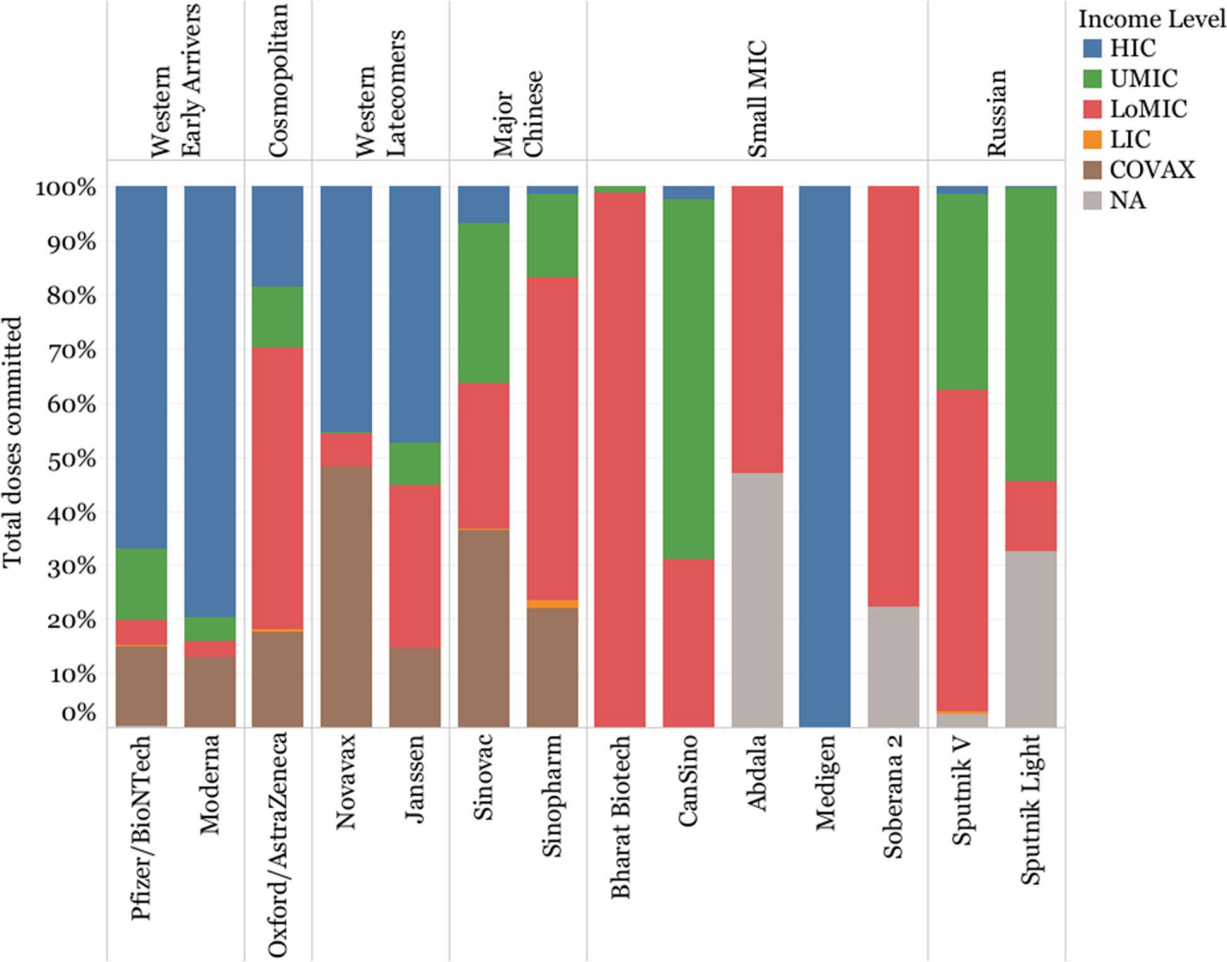
Share of vaccines committed by income level of purchasing country

Sinopharm and Sputnik V had a larger number of purchase agreements with LoMICs (59.68% and 59.64% respectively) over UMICs (15.50% and 36.17% respectively), whereas Sinovac seemed to follow a different strategy, with 36.67% of its doses dedicated to COVAX and 29.56% to UMICs. Oxford/AstraZeneca committed roughly half of its doses to LoMICs (52.14%), mostly to India, with the rest of the doses being roughly equally distributed between HICs (18.57%) and COVAX (17.57%), with UMICs receiving a smaller share (11.27%). Oxford/AstraZeneca accounted for the largest proportion of doses committed to LICs (18.5 million doses, 52.13% of all doses purchased by LICs), followed by Sinopharm (11.2 million doses, 31.54%) (Fig. 9).

**Fig. 10:**
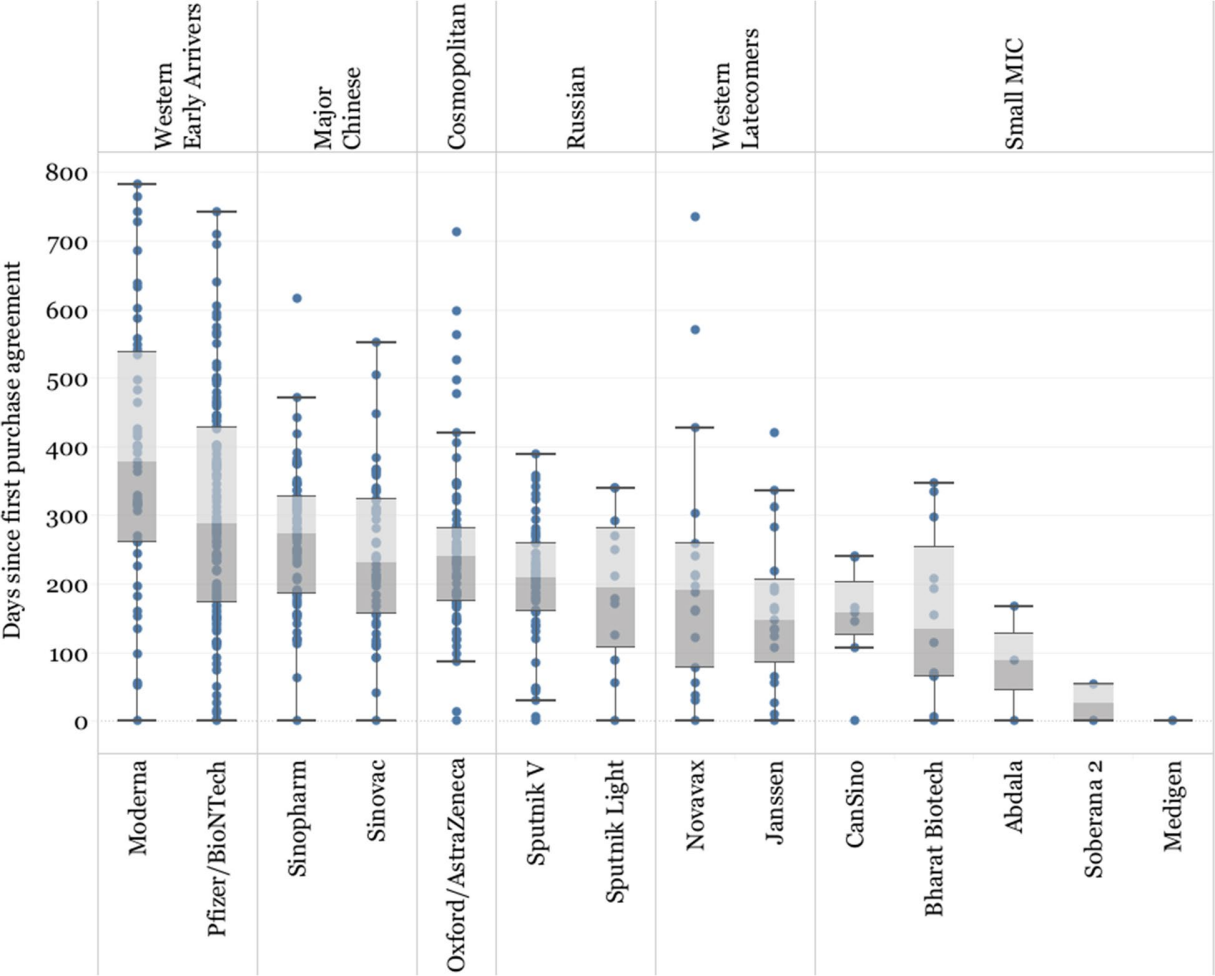
Time distribution of vaccine agreements. Axis Y shows the number of days since the first purchase agreement signed by each vaccine developer

When looking at the time distribution of purchase agreements, Pfizer/BioNTech and Moderna sustained a high number of agreements consistently throughout the period of analysis, until 750 and 800 days, respectively, after their first agreement was signed (Fig. 10), showing the sustained demand and market dominance of Western Early Arriver vaccines over other vaccine developers.

Oxford/AstraZeneca and the Major Chinese developers also have a wide distribution of agreements, although most of their agreements were signed within the first year following their first purchase agreement. Western Latecomers, Russian Developer, and the Small MIC Developers signed very few agreements after the first year following their first agreement.

### Vaccine deliveries

**Fig. 11:**
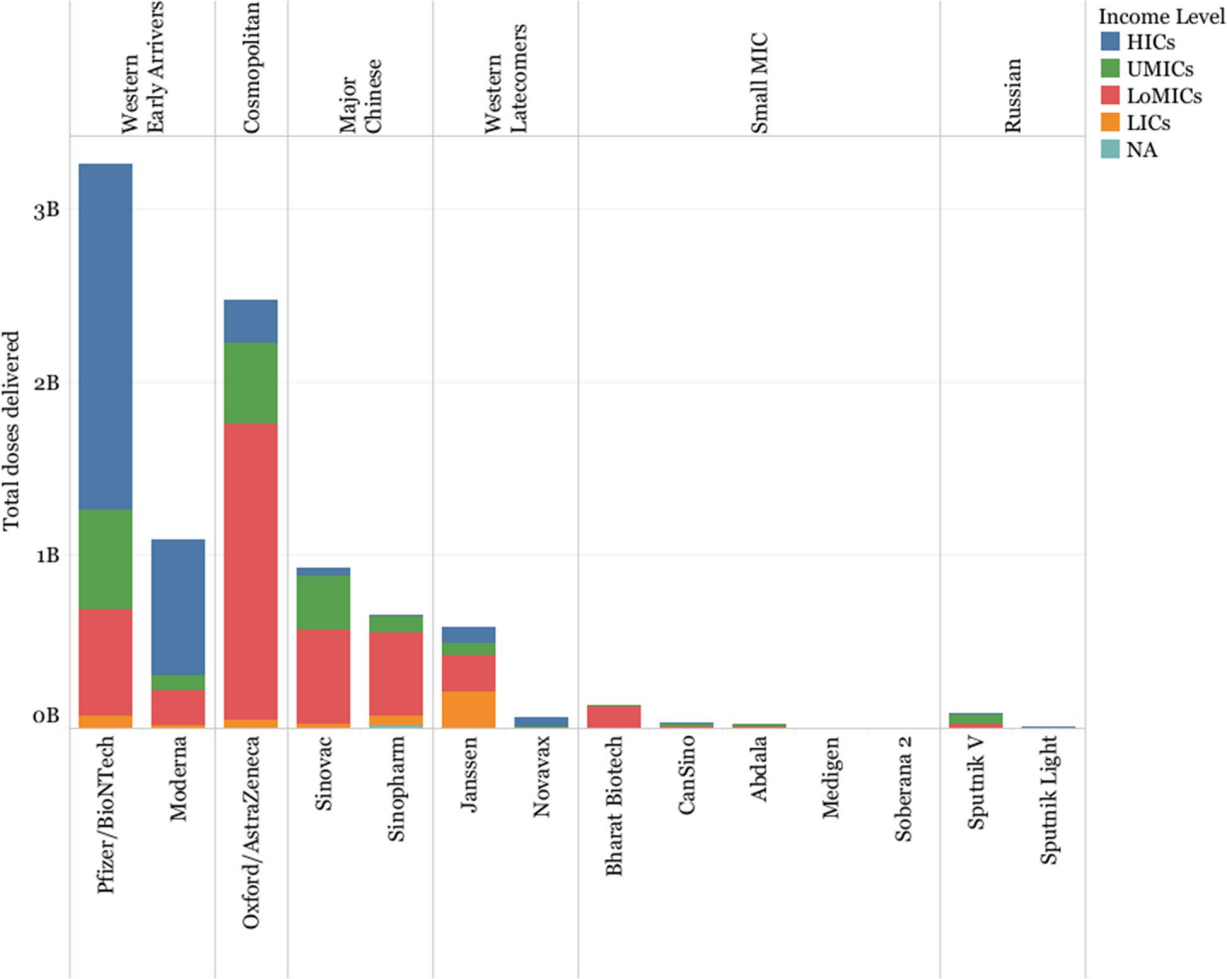
Doses by income group of the recipient country

Pfizer/BioNTech and Oxford/AstraZeneca have delivered more vaccine doses than other developers (Fig. 11), followed by Moderna, Sinovac and Sinopharm. However, the number of doses of Sinopharm and Sinovac are likely substantially undercounted, given that the vaccines delivered in China have not been reported publicly (see Limitations and areas for further research). Western Early Arrivers delivered most of their doses in HICs (61.40% Pfizer and 72.49% Moderna), compared with Oxford/AstraZeneca and the Major Chinese developers that prioritized LoMICs above other income groups (69.02% Oxford/AstraZeneca, 73.25% million Sinopharm, and 59.08% Sinovac). Western Latecomers (particularly Novavax) and Russian Developer delivered fewer doses than initially committed. Janssen is among the developers with more deliveries in LICs (208 million doses, 35.57% of all its deliveries) and LoMICs (207 million doses, 35.45%), mostly through COVAX (48.64% of its deliveries to these countries were through the global platform).

The distribution of vaccine deliveries was staggered by income level, with HICs receiving more vaccines earlier than LoMICs and UMICs, and with LICs accessing very few doses much later than the rest.

**Fig. 12:**
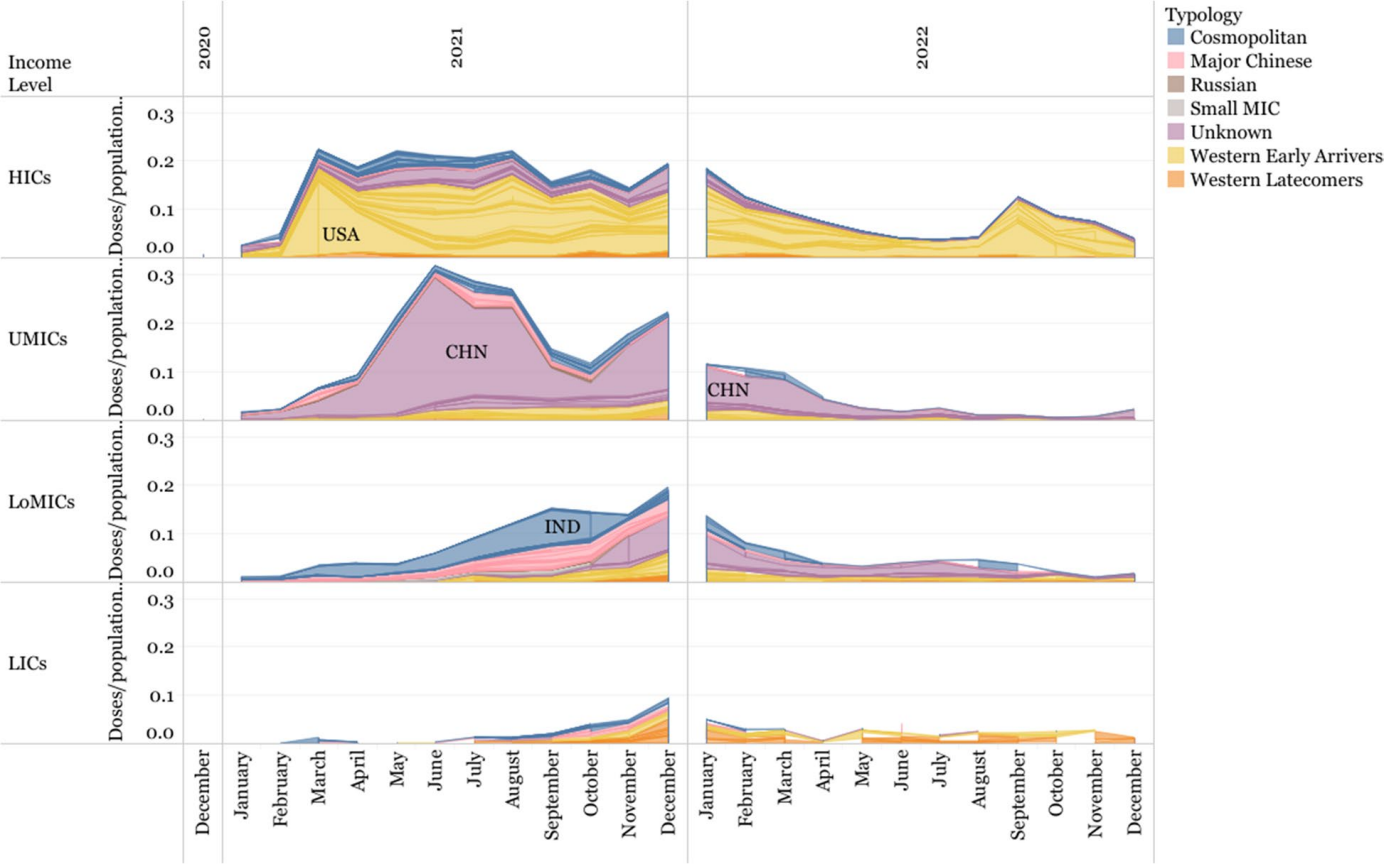
Timeline of vaccine deliveries for each innovation model and income level of recipient country. Doses are shown relative to the income group’s population for comparability

Deliveries in HICs occurred in two stages (Fig. 12): the first one peaked early in 2021 and remained constant throughout the rest of the year. The second stage was in 2022, where deliveries slowed down, perhaps due to a situation of market saturation, before rising again as the autumn began in the Northern Hemisphere and guidelines in many HICs - notably in the US, which accounts for much of the trend - recommended booster doses.

Deliveries in UMICs follow a pattern driven by China, which experienced two large peaks - one between April and September 2021, and another between December 2021 and January 2022, followed by a steep decline in 2022. However, it is not clear which vaccines were distributed in China, nor is it clear whether the lack of data for 2022 reflects a lack of vaccine distribution, or a lack of data on vaccine distribution within the country (Fig. 12). The drop in deliveries in China between August and December 2021 seems to correspond to an increase in exported deliveries from Major Chinese developer vaccines to LoMICs.

Deliveries from the Major Chinese Developers comprised a large proportion of supply in UMICs, and even more so in LoMICs, until late 2021. In LoMICs, there is a single, smaller peak between November 2021 and February 2022, with a large proportion of these deliveries being in India. The country had a large portion of deliveries of Oxford/AstraZeneca (manufactured by India-based Serum Institute) between June and October 2021 that could be related to the export bans imposed by the country from March to September 2021, to extend vaccinations nationally.

**Fig. 13:**
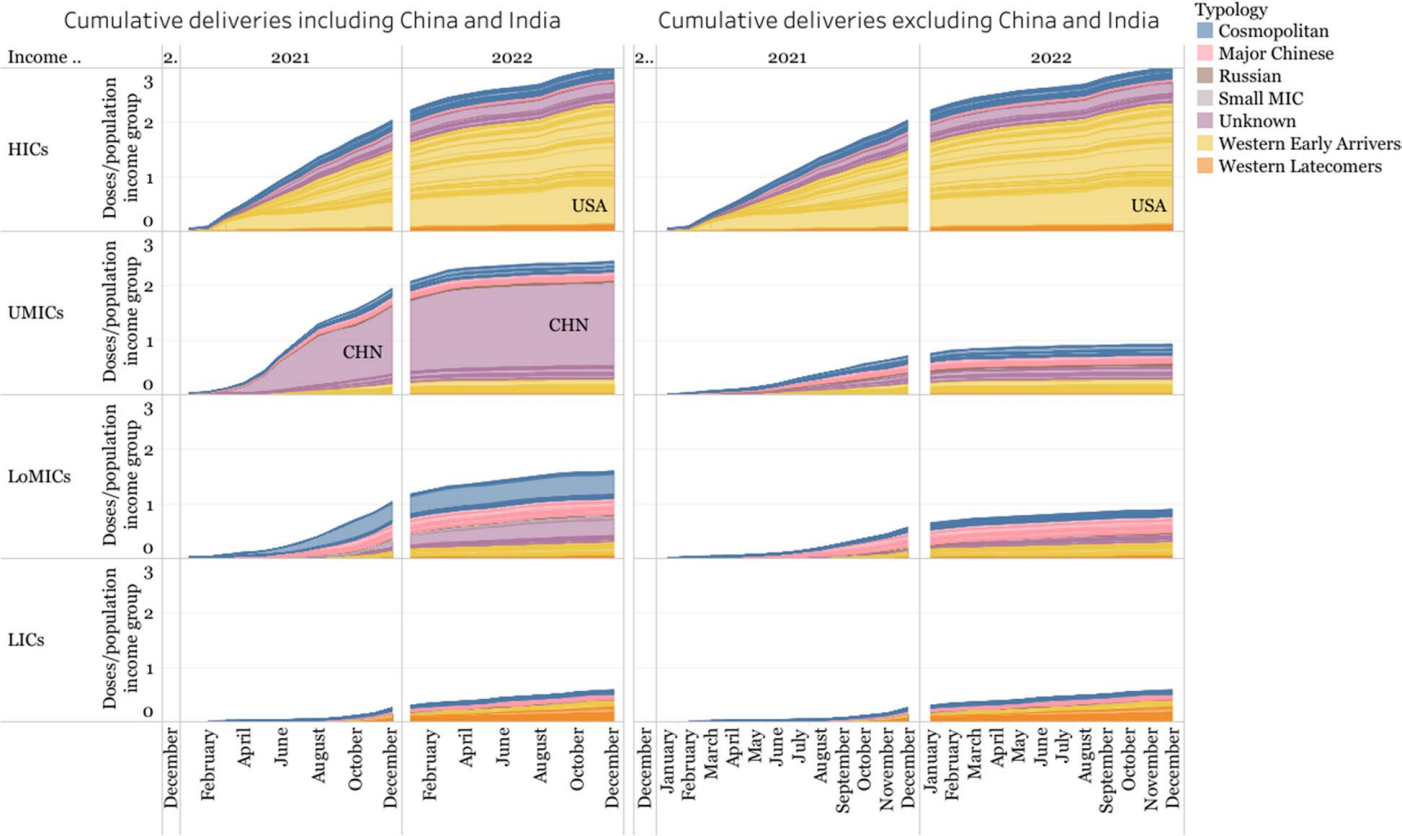
Cumulative deliveries relative to the population of the income group in 2021 and 2022. The left side includes all countries. Right side excludes deliveries in China and India

The pace at which UMICs and HICs accessed enough doses to vaccinate each person with one dose seems to be similar (Fig. 13, left side). Nevertheless, this is skewed by the large number of deliveries in China, which accounts for a large portion of the deliveries in UMICs. When removing China and India, the remaining deliveries in UMICs and LoMICs do not reach one dose per person in the period of analysis (Fig. 13, right side). Deliveries in LICs do not reach one dose per person in the entire period of analysis, demonstrating that access inequalities greatly affected this group of countries over others.

**Fig. 14:**
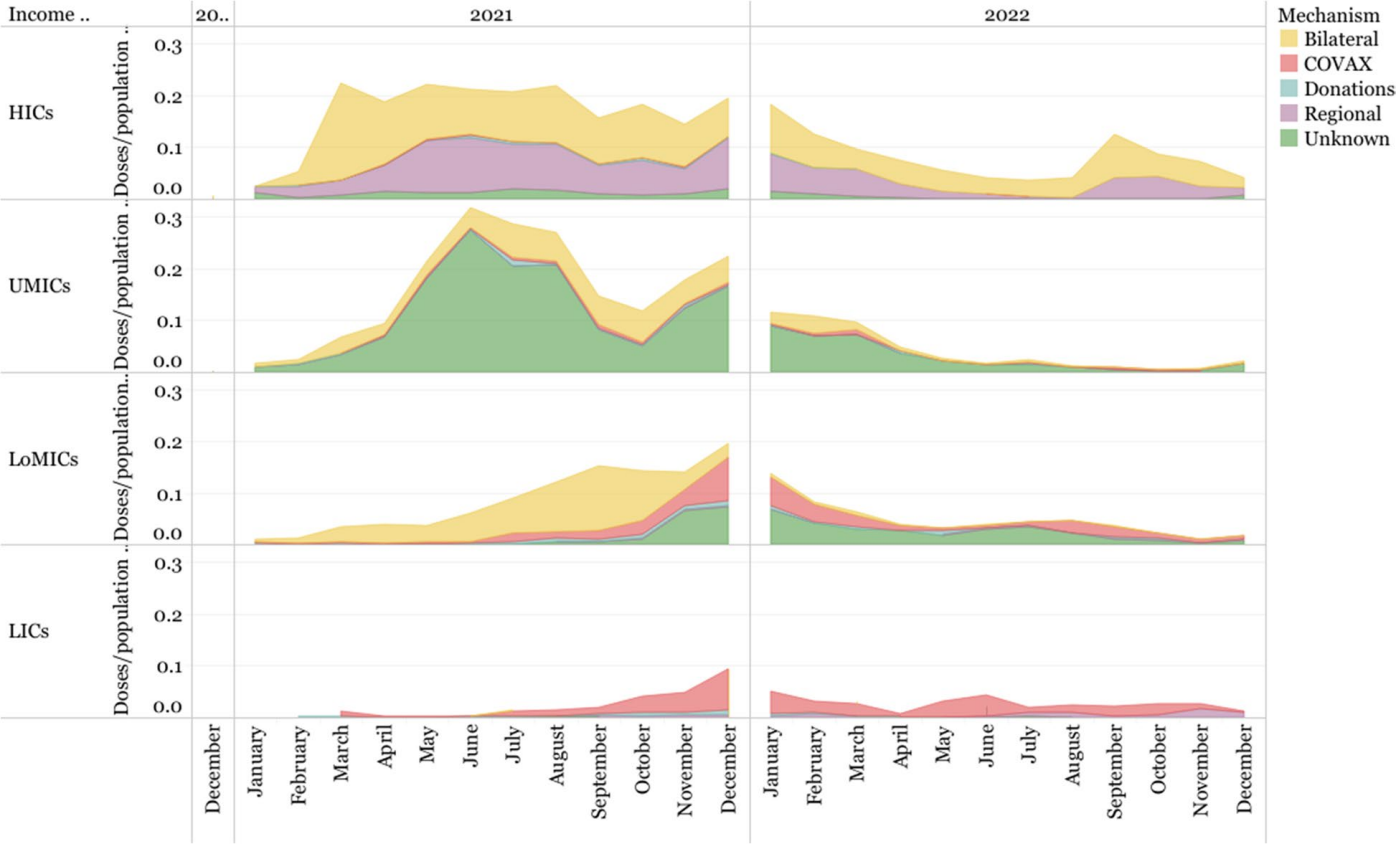
Vaccine deliveries by type of delivery mechanism

When looking at the channel chosen for the acquisition and delivery of vaccines, doses purchased through bilateral and regional (deliveries through the African Vaccine Acquisition Trust, AVAT) agreements with developers represent 45.80% of all deliveries, when all “Unknown deliveries” are included, and began earlier than the deliveries from COVAX, which represent 11.90% of all deliveries. In Fig. 14, deliveries via COVAX peak at the end of 2021, and were dedicated to LICs and LoMICs. Despite being the focus of substantial discussion and debate, donations represent less than 3% of all the deliveries. As mentioned in the Limitations and areas for further research section, a large share of the deliveries (39.34%) has no data on the delivery channel.

Small MIC Developers mainly delivered doses in their respective countries and regions of origin. Although there is no data on deliveries in Cuba, the two Cuban vaccines were used in the country according to publicly available information [111] and was exported to other Latin American countries (Mexico, Nicaragua, and Venezuela). Bharat Biotech and Medigen delivered most of their doses domestically (97.38% to India and 95.99% to Taiwan, respectively). Regarding CanSino’s deliveries, the percentage of doses delivered in China is not publicly availbale. Nevertheless, for doses outside of China, there is evidence that the company focused its deliveries on Mexico (50.87%) and Pakistan (38.55% of all its deliveries).

### Vaccine prices

Price information has not been systematically disclosed by developers or governments during the pandemic. Nevertheless, the data available shows that Oxford/AstraZeneca had the lowest average prices across the different income groups, ranging between 3.00 and 5.55 USD/dose. However, prices for HICs are lower than those for UMICs and LoMICs, although in some LoMICs such as India or Bangladesh, average prices are higher due to a different price for the private market. Similarly, Janssen, with an average price of around 10 USD/dose across income groups, also seemed to charge lower prices in HICs than in LoMICs and UMICs.

Moderna, Sinopharm, and Pfizer appear to have applied a tiered pricing approach, with clearly different prices for different income groups. The first two vaccines are also the two most expensive vaccines in HICs (40 and 36 USD/dose, respectively), and two of the most expensive vaccines in UMICs (28.88 and 19.98 USD/dose), although their LIC pricing is roughly on par with other developers selling to those markets. Pfizer’s tiered pricing is more of a binary split between pricing in HICs (20.85 USD/dose) and pricing in UMICs and LoMICs (11.2 and 10.58 USD/dose, respectively).

There are no price points in HICs for Sinovac and CanSino (Bharat Biotech did not deliver doses in HICs), and their listed prices for UMICs and LoMICs are higher than most of the other vaccine developers ranging from 15 to 27 USD/dose. Gamaleya, which committed to sell Sputnik V at less than 10 USD/dose [112], was reportedly selling it at almost 20 USD/dose in HICs and LoMICs, according to the data analyzed.

Despite orienting their operations towards supplying MICs, developers such as Sinopharm, CanSino, Gamaleya, Sinovac, and Bharat Biotech priced their products higher in these countries than other developers like Pfizer, Janssen, and Oxford/AstraZeneca.

**Fig. 15:**
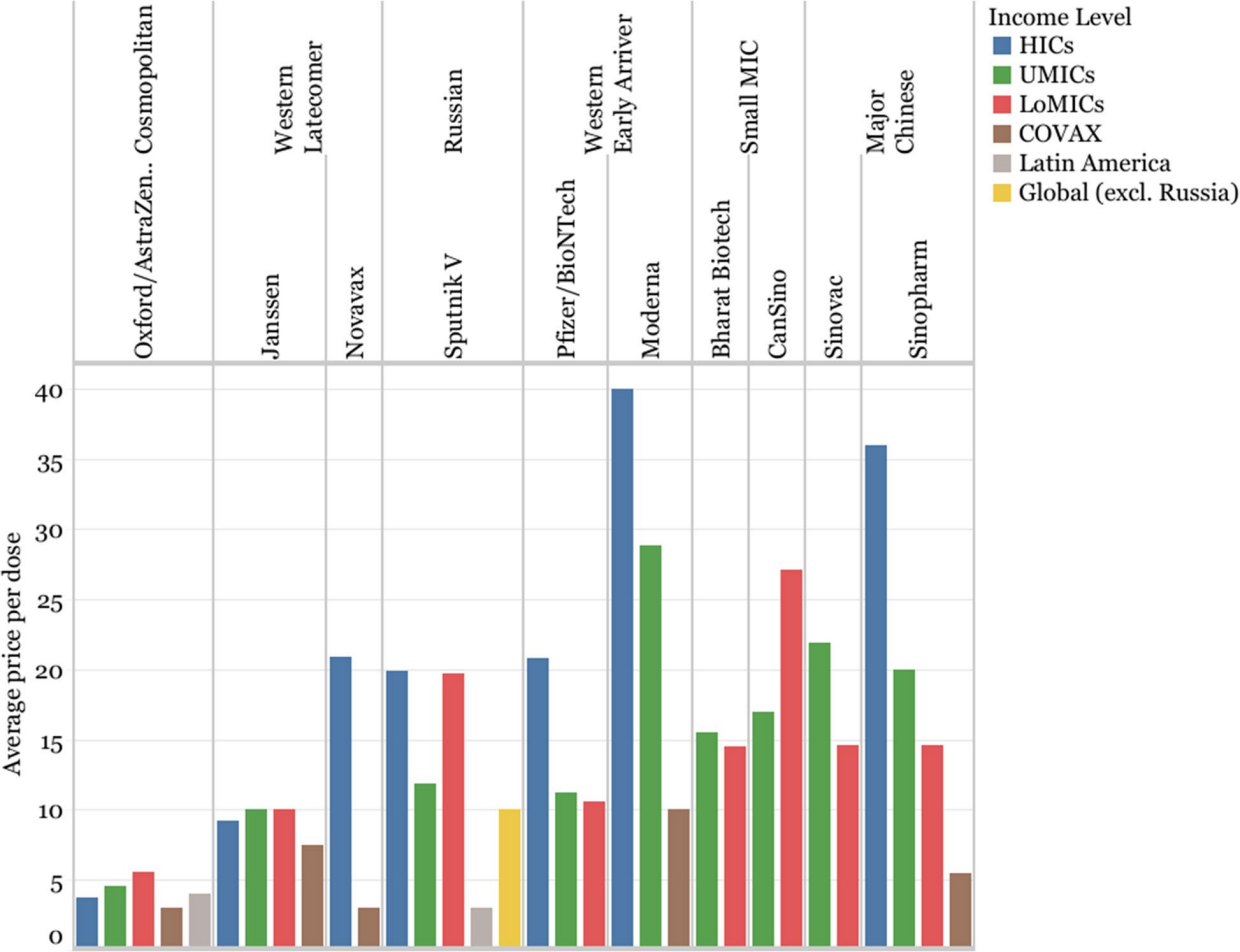
Average prices per dose per vaccine by income level

Prices available for COVAX are on average lower than for all income groups, ranging from 3 USD/dose (Oxford/AstraZeneca and Novavax), to 10 USD/dose (Moderna). Nevertheless, in the publicly-disclosed contract between COVAX and the South African government, the maximum vaccine cost is listed to be 21.10USD/dose, which is more than the price reported by COVAX [113] (Fig. 15).

## Discussion

### Strengths and weaknesses of COVID-19 vaccine innovation models

Our analysis shows that COVID-19 vaccine developers adopted a range of strategies to commercialize their vaccines, which can be roughly grouped into six innovation models that had varying strengths and weaknesses in addressing global access to vaccines during the pandemic.

Western Early Arrivers received substantial public financial support from Western governments, especially from the US and the European Union, which arguably accelerated their development and gave them a head start in the market. These vaccines were among the first to be commercialized through APAs, furthering their market dominance. In addition, these vaccines showed very high efficacy results in clinical trials, and despite the logistical problems related to storage and cold chain requirements for mRNA vaccines, the global demand was very high (as evidenced by the large number of purchase agreements). Both developers (but particularly Pfizer/BioNTech) showed strong capacity to obtain regulatory approvals, sign purchase agreements, and deliver doses globally. This suggests that, among all developers, they had the greatest capacity to supply all countries. Nevertheless, both developers prioritized their activities in HICs before supplying LoMICs and UMICs, and have one of the lowest percentages of deliveries in Low Income Countries (1.93% of all the model’s deliveries, 84.13 million doses). Despite obtaining WHO Emergency Use Listing (EUL) and accounting for roughly 40% of all doses delivered through COVAX (Pfizer/BioNTech represents 31.76% of COVAX’s deliveries, and Moderna 9.92%), only 18% of all the doses delivered by this model were done through COVAX.

On the other hand, by partnering with a large pharmaceutical company (AstraZeneca), Oxford University leveraged the ‘material power’ of the pharmaceutical industry - its control of global supply chains, production capacity, and clinical trial development [114]. This, among other factors (e.g., type of technology, tech transfer/manufacturing agreement with SII), allowed them to achieve relatively more widespread access to its vaccine across income groups. Since the technology used (adenoviral vector) had lighter cold chain and storage requirements than mRNA vaccines, and since most HICs shifted their purchasing priorities towards other vaccines, this vaccine was well positioned to address global needs.

The licenses with AstraZeneca and later with Serum Institute of India showed relatively successful results in terms of access in LoMICs and UMICs. It quickly obtained regulatory approval in the largest number of countries of any of the vaccines analyzed, with no major distinctions between HICs, UMICs, and LoMICs; and was able to supply more doses to L&MICs than any other developer. It accomplished this through a dual strategy: AstraZeneca focused on HICs and UMICs, and the Serum Institute of India commercialized the vaccine in L&MICs, with a particular focus in India. The 2021 export ban in India seems to have limited supply to other L&MICs, highlighting the risk of relying too heavily on one partner manufacturer, but an in-depth exploration of the export ban’s impact is outside the scope of this research. The vaccine seems to have been priced lower than most of the other vaccines in the group, although prices seem to be higher in L&MICs than in HICs. Despite this, only 2.04% of all its doses were delivered in LICs (50.4 million doses), showing a big limitation addressing global access.

Western Latecomers also received large sums of public funding to advance the development process, and in the case of Novavax, the company was also supported by CEPI. The scale of operations of these two vaccine developers is smaller than the Western Early Arrivers and Cosmopolitan models, especially in Novavax’s case. Janssen’s initial dosing regimen consisted of a single dose, which could have translated to a more efficient purchasing, delivery, and administration strategy, making it particularly well suited for use in L&MICs, despite the cold-chain requirements. Similarly, Novavax’s protein subunit technology had less extreme storage and cold-chain requirements than mRNA and viral vector vaccines, and was a well-known and longstanding technology, potentially being better suited for use in resource-limited settings [104]. These factors, among others (e.g., availability of doses), may have shaped the demand for these vaccines in L&MICs, shown by Janssen’s purchase agreements with the African Union and COVAX, or Novavax’s agreement with COVAX, becoming the model with more deliveries in LICs (208.2 million doses, 32.33% of all its deliveries). Nevertheless, Janssen’s dosing regimen changed after showing higher efficacy with two doses, and Novavax experienced manufacturing and regulatory challenges and delays that prevented the company from meeting its targeted deliveries [115], limiting global access.

Our analysis shows the increasing role and capacity of non-Western actors in the global pharmaceutical innovation system. Major Chinese Developers, Sinopharm and Sinovac, prioritized LoMICs and UMICs to obtain regulatory approvals, sign purchase agreements, and deliver their vaccines, often being the first to do so among vaccine developers in many of these countries. The platform used by these developers (whole virion inactivated) is well known, and although the efficacy of both vaccines was reported as lower than mRNA and viral vector vaccines, they had less demanding storage and cold chain specifications, making them easier to deploy in resource-limited settings.

Both vaccines entered the market early, obtained WHO EUL, and provided doses to COVAX, although in lower volumes than both Western models and Oxford/AstraZeneca. In addition, only 4.5% of the model’s doses (71.2 million) were delivered to LICs. The amount of public sector investment in the development of these vaccines is unclear, however, the Chinese public sector played a large role in coordinating efforts and striking partnerships between public and private organizations, as well as in coordinating administration within China [63], although detailed data are unavailable.

The Russian vaccines (both developed by Gamaleya) faced many challenges with production and supply, which clearly influenced their commercialization strategy. Sputnik V was the first globally approved vaccine, and aimed to be available worldwide, prioritizing UMICs and LoMICs. Due to limited in-house production capacity, Gamaleya sought to create a wide network of manufacturing partners for end-to-end production, particularly in UMICs and LoMICs [102]. However, production scale-up issues, quality problems, and delivery delays undermined this strategy, likely contributing to the very low number of doses delivered worldwide [102]. Gamaleya sought WHO EUL, but quality concerns during inspections stopped the process until recently, and therefore they have not been able to sign purchase agreements with COVAX [80, 102]. The extent of public sector investment seemed to be large given the substantial public subsidies obtained by the public research center and the involvement of the Russian sovereign investment fund (RDIF), although its influence on Gamaleya’s practices is unclear.

Small Middle-Income Country Developers are a relatively more heterogeneous group, but they share some common characteristics. These vaccines had a limited global presence, many entering late an already saturated market and most had lower global production capacity. These vaccines were supported financially by their respective governments, and for those where there is data available, were overwhelmingly used to support national vaccination efforts. Most of these developers were based in MICs, used well-known technologies that had lighter storage and cold chain requirements than the mRNA vaccines, and reported a wide range of vaccine efficacies. Although most vaccines in the group applied to obtain WHO EUL (all except Soberana), only CanSino managed to supply doses to COVAX after Bharat Biotech’s supply agreement was canceled [116]. Arguably, the emergence of these developers could have the potential to influence future pandemic response efforts by filling unmet needs in their countries, regions and beyond, even if the capacity of any of these developers to ensure global access is currently quite small. Nevertheless, their influence could potentially be magnified in the future, for example, by expanding collaborative networks to leverage their combined scientific, regulatory, and market expertise, and/or increasing their current production capacities.

As Suzuki and Yang [64] put it “[t]he COVID-19 pandemic marked the debut of non-Western powers as vaccine inventors on the world stage”. This emergent geographic diversity, along with the organizational diversity of vaccine developers, tentatively challenge what Kapczynski calls the ‘ideational power’ of the (Western) pharmaceutical industry. This term describes the assumption that there is an “inability of anyone but the private sector to innovate, and a skepticism about government power” [114]. Although academic institutions, state-owned companies, but also for-profit companies located in MICs, showed capacity to develop and deploy COVID-19 vaccines globally, these models are still emergent and their impact is smaller in scale and capacity compared to Western early arrivers or Oxford/AstraZeneca. At the same time, the profit motive appears to remain salient across most models, as reflected in the (limited) pricing data available and the limited supply to Low Income Countries.

### The role of public institutions in shaping developers’ practices

Public funding, actors and policies, among other factors, shaped vaccine development and commercialization. All developers analyzed received public support through different policy instruments that covered the entire development process. Public investments pushed all developers except two, to later stages of development, in some cases, seemingly funding the entire development process. APAs pulled the development of primarily Western Early Arrivers and to a lesser extent Western Latecomers and Oxford/AstraZeneca by de-risking the expansion of manufacturing capacity and late-stage clinical trials, signaling demand size and prioritizing access in certain countries. As pointed out by Florio and collaborators [117], taxpayers were “major funders of corporate R&D and productive investment”. Governments also played a pro-active role beyond funding, prioritizing vaccine technologies and facilitating development, such as in the case of the Chinese and US government’s efforts to coordinate the development of vaccines [63, 118, 119], or in the case of the US NIH providing in-kind support to Medigen [106].

The use of advance purchase agreements by HICs to secure national access captured the global supply of Western Early Arrivers’ vaccines, thereby generating global inequalities in access to these vaccines. In addition, the limited data available indicates that large MICs with industrial and innovative capacity (i.e. China, Russia or India) also seem to have prioritized vaccine access for their own citizens first, before engaging in substantial exports.

Other, usually smaller MICs used their R&D capacity to develop and deliver vaccines nationally and regionally. The data in this analysis show that LICs were at the end of the line, unable to access enough vaccines through direct purchase agreements, and left queuing for the limited supply available to COVAX, which was substantially slower than vaccine access via other mechanisms.

### The role of global health initiatives

CEPI and COVAX, the two global health initiatives mandated to support global access to COVID-19 vaccines, influenced many of these innovation models. Many of CEPI’s investments led to the development of vaccines (i.e. Novavax, Medigen, Oxford University, Moderna), underscoring the advantages of having independent R&D coordinators and funders with a global view of the technological landscape. Nevertheless, CEPI’s access principles had limited success, as they were “affected by governments entering agreements or asserting other legal claims affecting CEPI-covered agreements”, ultimately jeopardizing CEPI’s ability to achieve its global access objectives [120]. Oxford/AstraZeneca is a relative outlier among recipients of CEPI’s investments, in terms of its impact expanding global access. Further research is needed to explore the replicability of Oxford/AstraZeneca’s strategy, the benefits and limitations of its licensing model, and the role of academic institutions as access-oriented developers [58].

COVAX made arrangements with all Western Early Arrivers, Latecomers and AstraZeneca (in Novavax’s case, the arrangement was terminated in late 2022); as well as with the two Major Chinese Developers, CanSino and Bharat Biotech, although Bharat Biotech’s supply was suspended due to quality concerns [116], but the timing and quantities acquired and delivered varied greatly. All Western developers (including Oxford/AstraZeneca) except Novavax obtained WHO EUL within the first 4 months of 2021, and subsequently contributed to COVAX. In contrast, the four non-Western developers (Bharat Biotech, Sinopharm, Sinovac, and CanSino) obtained EUL in the second half of 2021, and contributed fewer doses than the Western developers. Our analysis also shows that deliveries through COVAX happened later than many bilateral and regional agreements.

## Conclusion

Our analysis illustrates the diversity of actors, countries, and practices surrounding the development of vaccines against COVID-19. Contrary to the monolithic treatment that vaccine developers seem to receive in the literature, there is not a single innovation model that all vaccine developers adopted during the global health emergency. Each model had its own particularities, and affected global supply of vaccines in different ways. Additionally, our analysis also shows the oftentimes unrecognized industrial and innovative capacity that resides in Middle-Income Countries.

Despite this, Low Income Countries made disproportionately fewer purchases, received fewer doses, at later dates, than MICs or HICs, and were directly affected by COVAX’s challenges to deliver doses in a timely and sufficient manner.

The political reality of the response during the COVID-19 pandemic shows the prioritization of national or regional access to vaccines before global commitments. The lack of a common legal framework to deliver global access hinders the capacity to leverage the increasingly globalized and diverse pharmaceutical R&D ecosystem to reduce access inequities. Such a legal framework is currently being considered in the negotiations towards a WHO Pandemic Accord and in amendments to the International Health Regulations. An equitable legal framework could facilitate the transfer and/or pooling of technology and knowledge to accelerate innovation and expand production, including through the inclusion of access conditions in R&D investments. It could also increase developers’ freedom to operate and legal certainty by protecting them from IP litigation on key technologies needed for emergencies.

As the likelihood of new outbreaks and global emergencies increases, and industrial and R&D capacities become progressively distributed worldwide, governments have strong incentives to harness its potential, and collaborate to ensure a more equitable and effective response in the future.

### Supplementary Information

**Additional file 1.** Database from UNICEF Market Dashboard containing data on regulatory approvals for all vaccines included for analysis (https://www.unicef.org/supply/covid-19-vaccine-market-dashboard).

**Additional file 2.** Database from UNICEF Market Dashboard containing data on vaccine prices for all vaccines included for analysis. The data can be downloaded as well in UNICEF’s website (https://www.unicef.org/supply/covid-19-vaccine-market-dashboard).

**Additional file 3.** Database from UNICEF Market Dashboard containing data on production agreements for all vaccines included for analysis. The data can be downloaded as well in UNICEF’s website (https://www.unicef.org/supply/covid-19-vaccine-market-dashboard).

**Additional file 4.** Database from UNICEF Market Dashboard containing data on purchase agreements for all vaccines included for analysis. The data can be downloaded as well in UNICEF’s website (https://www.unicef.org/supply/covid-19-vaccine-market-dashboard).

**Additional file 5.** Database from UNICEF Market Dashboard containing data on monthly vaccine deliveries for all vaccines included for analysis. This database was provided by the UNICEF team, but the data can be obtained in UNICEF’s website (https://www.unicef.org/supply/covid-19-vaccine-market-dashboard).

**Additional file 6.** Database from the Global Health Centre (GHC)’s Knowledge Portal on Innovation and Access to Medicines (https://www.knowledgeportalia.org/covid-19-vaccine-r-d-funding), containing data on R&D investments tracked between 2020 and 2021 for all vaccines included for analysis.

## Data Availability

The datasets supporting the conclusions of this article are included in the article and its additional files. Furthermore, the original dataset on R&D investments can be accessed in the Global Health Centre’s Knowledge portal on innovation and access to medicines (https://www.knowledgeportalia.org/covid-19-vaccine-r-d-funding) and the rest of the data can be downloaded in UNICEF’s COVID Market Dashboard (https://www.unicef.org/supply/covid-19-vaccine-market-dashboard).

## Footnotes

1 See Limitations and areas for further research.
